# Perspectives of future lung toxicology studies using human pluripotent stem cells

**DOI:** 10.1007/s00204-021-03188-9

**Published:** 2022-01-01

**Authors:** Atsushi Masui, Toyohiro Hirai, Shimpei Gotoh

**Affiliations:** 1 Department of Drug Discovery for Lung Diseases, Graduate School of Medicine, Kyoto University, Kyoto, Japan; 2 Watarase Research Center, Kyorin Pharmaceutical Co. Ltd., Shimotsuga-gun, Nogi, Tochigi Japan; 3 Department of Respiratory Medicine, Graduate School of Medicine, Kyoto University, Kyoto, Japan

**Keywords:** Lung, Airway, Alveolar, Pluripotent stem cell, iPS cell, Organoid

## Abstract

The absence of in vitro platforms for human pulmonary toxicology studies is becoming an increasingly serious concern. The respiratory system has a dynamic mechanical structure that extends from the airways to the alveolar region. In addition, the epithelial, endothelial, stromal, and immune cells are highly organized in each region and interact with each other to function synergistically. These cells of varied lineage, particularly epithelial cells, have been difficult to use for long-term culture in vitro, thus limiting the development of useful experimental tools. This limitation has set a large distance between the bench and the bedside for analyzing the pathogenic mechanisms, the efficacy of candidate therapeutic agents, and the toxicity of compounds. Several researchers have proposed solutions to these problems by reporting on methods for generating human lung epithelial cells derived from pluripotent stem cells (PSCs). Moreover, the use of organoid culture, organ-on-a-chip, and material-based techniques have enabled the maintenance of functional PSC-derived lung epithelial cells as well as primary cells. The aforementioned technological advances have facilitated the in vitro recapitulation of genetic lung diseases and the detection of ameliorating or worsening effects of genetic and chemical interventions, thus indicating the future possibility of more sophisticated preclinical compound assessments in vitro. In this review, we will update the recent advances in lung cell culture methods, principally focusing on human PSC-derived lung epithelial organoid culture systems with the hope of their future application in toxicology studies.

## Introduction

**Fig. 1:**
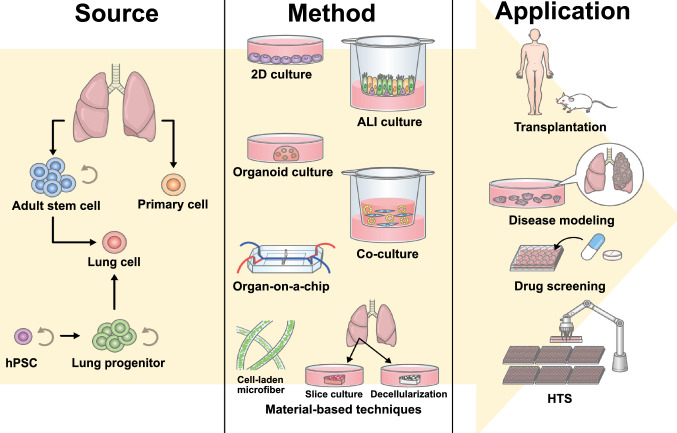
Overview of lung cell sources, culture systems, and their applications. Major cell sources of human lung cells are primary cells from lung tissue, PSC-derived lung progenitor cells and their differentiated airway and alveolar epithelial cells. A variety of culture methods have been used for achieving cell maturation and demonstrating their functions. These culture methods are used for disease modeling and drug screening. The ultimate goal is to utilize them for high-throughput screening (HTS) and transplantation therapy for the lung diseases of unmet medical needs

Researchers have reported on drug-induced lung injury for therapeutic agents with various points of action, including cytotoxic and non-cytotoxic chemicals (Cooper et al. 1986a, b; Rossi et al. 2000; Camus et al. 2004; Jo et al. 2021). Bleomycin (BLM), a clinical anticancer drug, causes drug-induced lung injury with an approximate frequency of 46% (Sleijfer 2001). BLM-treated mice have been widely used as animal models of idiopathic pulmonary fibrosis (Moeller et al. 2008). Several cytotoxic anti-tumor drugs, including BLM, can cause drug-induced lung injury (Rossi et al. 2000; Camus et al. 2004). In addition, lung injury side effects have also been reported for molecularly targeted agents and immune checkpoint inhibitors, which are currently under active research and development (Inoue et al. 2003; Kudoh et al. 2008; Rizvi et al. 2015; Garon et al. 2015). Furthermore, the incidence of drug-induced lung injury caused by gefitinib, a selective epidermal growth factor receptor (EGFR) inhibitor, varies according to ethnicity (Kudoh et al. 2008; Shi et al. 2014). The risk of drug-induced lung injury can cause the withdrawal of an effective drug, resulting in clinical problems as well as large economic losses (DiMasi et al. 2016). The ICH Safety Assessment Guideline (S7A) enlists a safety assessment of the respiratory system as a top priority, which is important for maintaining vital functions. It could be of great clinical benefit to assess the risk of drug-induced lung injury at the drug development stage. However, there are limited useful tools for predicting adverse drug reactions in the respiratory tract. It is difficult to culture primary lung cells while maintaining their characteristics in vitro; therefore, immortalized cell lines have been used for in vitro studies. However, these cell lines have a limited ability to mimic the response of normal lung cells. For example, A549, which is used as a type 2 alveolar epithelial cell line, does not express lineage-specific markers of NKX2.1 and surfactant proteins. Its reactivity to lipopolysaccharide (LPS) or cytokines reportedly varies from that of primary alveolar epithelial cells (Witherden et al. 2004; Kolla et al. 2007). In contrast, the cells can mimic the phenotype of type 2 alveolar epithelial cells via gene modification. Kanagaki et al. reconstructed lamellar body (LB)-like organelles in A549 cells exogenously expressing surfactant protein genes. Those “LB cells” were able to simulate the secretion of surfactant proteins (Kanagaki et al. 2021b), and were useful in evaluating the abnormalities of lamellar bodies and identifying a potential therapeutic agent. Microfluidic technology has contributed to recapitulating the pulmonary vascular barrier in the co-culture of endothelial cells and A549 cells on a chip (Huh et al. 2010). Mice and other laboratory animals have been useful for in vivo studies, despite the limitations associated with species differences, such as different lung structures and varied responsiveness to drugs (Moeller et al. 2008; Matute-Bello et al. 2008). The limitation is that the aforementioned approaches could recapitulate only a part of the type 2 alveolar epithelial cells. In this context, researchers have made remarkable recent developments in human-derived lung cell culture systems (Fig. 1). Organoid culture techniques for human primary cells (Barkauskas et al. 2017) and the methods of generating human pluripotent stem cell (PSC)-derived lung cells (Huang et al. 2013; Gotoh et al. 2014; Dye et al. 2015; Hawkins et al. 2017) have enabled the use of lung lineage cells that closely resemble biological functions for in vitro assays. The combination of lung-on-a-chip technology and human primary/PSC cells will likely be used in functional evaluation (Gkatzis et al. 2018). The exploration of various cell culture methods has led to their active use in diverse disease models and drug assessments (Table 1). In this review, we will introduce the recent technologies of lung-related cell culture systems, including human PSC-derived cells, and discuss their use for a toxicity assessment of drug-induced lung injury along with efforts in other organs.

**Table 1:** Milestones in the application of human PSC-derived cells or organoids

	Human PSC-derived lung cell culture	Application	References
2011	Generation of anterior foregut endoderm from PSC		Green et al. (2011)
2012	Airway epithelial cells	Cystic fibrosis (CF)	Wong et al. (2012)
2013	Lung epithelial cells		Huang et al. (2013)
2014	Multiciliated cells	CF	Firth et al. (2014)
	Fibroblast-dependent alveolar spheroid		Gotoh et al. (2014)
2015	Lung organoid		Dye et al. (2015)
2016	Airway organoid		Konishi et al. (2016)
2017	Airway organoid	CF	McCauley et al. (2017)
	Lung bud organoid	Hermansky–Pudlak syndrome type I Respiratory syncytial virus (RSV) infection	Chen et al. (2017)
	Alveolar spheroid/organoid	Surfactant protein B deficiency Drug toxicology	Jacob et al. (2017) Yamamoto et al. (2017)
2018	Lung tip organoid	Engraftment into mouse airway	Miller et al. (2018)
2019	Isolation of AT2 cell using SLC34A2	Hermansky–Pudlak syndrome type II (HPS2)	Korogi et al. (2019)
2020	Alveolar cells	SARS-CoV-2 infection	Huang et al. (2020)
	Alveolar organoid	SARS-CoV-2 infection	Han et al. (2020)
2021	Airway basal cell organoid	Asthma, CF and primary ciliary dyskinesia (PCD)	Hawkins et al. (2021)
	Airway cells	SARS-CoV-2 infection	Yin et al. (2021)
	Airway cells in airway-on-a-chip	PCD	Sone et al. (2021)
	Microfiber-based expansion of lung progenitor cells	Engraftment into mouse alveoli	Ikeo et al. (2021)

### Cardiomyocytes

The use of PSC-derived cells for drug toxicity studies has been intensive in the cardiovascular systems. The ICH safety pharmacology studies for human pharmaceuticals guidelines (S7A) recognize the evaluation of the effects on the respiratory and cardiovascular systems of all drugs under development as one of the most important issues. An appropriate assessment of the effects of compounds on the cardiovascular system is mandated in the Safety Guideline (S7B) of ICH. Drug-induced QT interval prolongation and the consequent occurrence of lethal arrhythmia Torsade de Pointes (TdP) is a significant clinical risk (Roden 2004). The conventional screening of drug candidates involves evaluating their effects on hERG channels and the assessment of electrocardiograms in animals to identify compounds at a high risk of causing lethal arrhythmias. However, compounds with strong inhibitory effects on hERG channels do not necessarily induce TdP, and it is not always possible to accurately evaluate the risk (Lu et al. 2008; Doherty et al. 2013; Morissette et al. 2016; Pollard et al. 2017; Park et al. 2018; Leishman et al. 2020). Researchers have attempted to use human PSC cell-derived cardiomyocytes to assess the pro-arrhythmic risk (Ando et al. 2017; Blinova et al. 2018). Enrichment with cell surface markers and tissue reporter cells enables the production of cells of sufficient quality and quantity for large-scale screening. In addition, a major trend is emerging in the use of PSCs in cardiotoxicity evaluation studies, as evidenced by the 2018 CiPA scientific workshop attended by scientists, clinicians, and regulators from multiple regions to discuss the utility of human PSC-CM in assessing the pro-arrhythmic risk of drugs using cardiomyocytes (Strauss et al. 2018). Moreover, the development of scalable culture methods that can provide a stable source of cells and the ability to reproduce the clinical phenotype of a beating heart at the cellular level have contributed to the popularity of drug evaluation applications.

### Hepatocytes

Of all clinical trials dropped out because of adverse effects or toxicity in drug development, drug-induced liver injury (DILI) has an incidence of approximately 20% (Kaplowitz 2005). DILI is broadly classified as intrinsic and idiosyncratic. In both cases, screening compounds at the preclinical stage with high sensitivity and specificity provides significant benefits to both patients and pharmaceutical companies. In vitro systems have been extensively developed as a tool for the assessment of DILI, such as 2D monolayer and 3D cultures using cell lines or primary cells, organoids, co-cultured hepatocytes and non-parenchymal cells, and organ-on-a-chip with options to link to other organ chips, because the conventional 2D culture does not adequately reflect the human hepatotoxic response. HepG2, a hepatocyte cell line, has improved drug metabolism and sensitivity to hepatotoxic drugs in 3D culture (Godoy et al. 2014). In addition, researchers have attempted the use of human PSC-derived hepatocytes (Lauschke et al. 2016). Recently, Takebe et al. reported on a high-throughput screening (HTS) system using human PSC-derived liver organoids (human liver organoids; HLO). The model does not include elements of the immune system; however, human PSC-derived cells were able to detect drug toxicity associated with genetic mutations (Shinozawa et al. 2021). The implementation of HTS using structurally and functionally complex HLO may be applicable to other organ fields. In the liver field, it has been possible to reproduce the 3D structure of organoids containing multiple cell types in vitro. Therefore, it is possible to construct an evaluation system that can predict the response to drugs in vivo. However, human PSC-derived hepatocytes are partially immature, and the distance from mature hepatocytes remains a common issue for PSC-derived cells.

### Primary cells

**Fig. 2:**
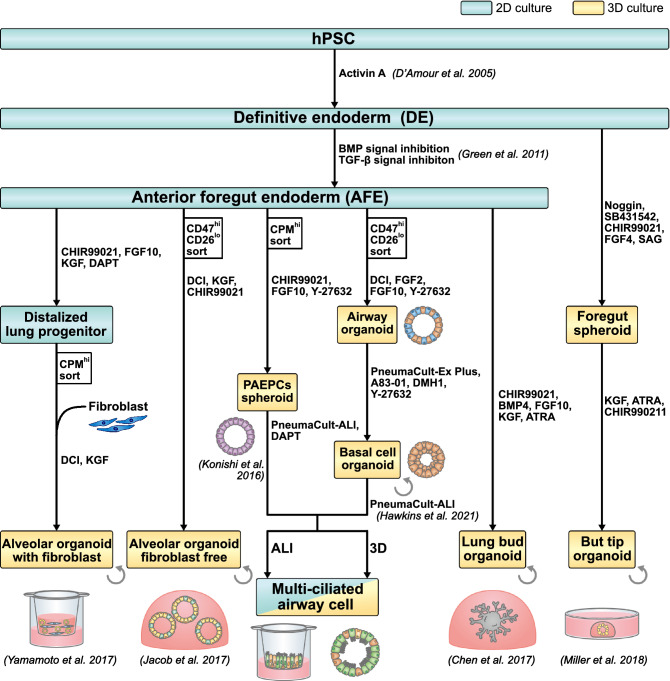
Schematics of the various methods of generating human PSC-derived lung epithelial cells. Human PSCs were differentiated into definitive endodermal cells using Activin A-based induction medium (D’Amour et al. 2005). Then, anterior foregut endodermal cells or foregut cell spheroids were induced. Matrigel-embedded self-assembled organoid culture was adopted in the most of 3D culture methods (orange), 2D culture means flat culture on plastic plates or air–liquid interface (ALI) culture on cell culture inserts (blue). The loop arrows represent capacity of expansion. There are two methods of directed induction of alveolar epithelial cells: fibroblast-dependent and fibroblast-free. In addition, there are two methods of generating airway epithelial progenitor cells: proximal airway epithelial progenitor cells (PAEPCs) and basal stem cells. Both cell types can be differentiated into ciliated cells and secretory cells in ALI and 3D culture. Generation of human PSC-derived lung organoids consisting of alveolar and airway regions is reported in two different manners: lung bud organoids and bud tip organoids

Since the 1987 report on 3D culture technology that enabled the culture of alveolar epithelial type 2 (AT2 or AEC2) cells isolated from rat lung tissue while maintaining their characteristics (Shannon et al. 1987), researchers have produced organoids from adult stem cells, mainly basal cells (Rock et al. 2009) and type 2 alveolar cells (Barkauskas, et al. 2017). Through the development of reporter mice or human PSC lines for each cell type, these organoid culture techniques have substantially contributed to our understanding of lung development and tissue repair mechanisms post-injury (Alysandratos et al. 2021b). Currently, accumulating reports have been published on the expansion and isolation of stem cells from primary lung tissue organoids (Mou et al. 2016; Sachs et al. 2019; Katsura et al. 2020; Youk et al. 2020; Salahudeen et al. 2020) as well as fetal lung tissue-derived ones (Nikolić et al. 2017; Miller et al. 2018). The aforementioned methods and applications will likely become more widespread in future. Organoids composed of primary cells supposedly comprise established culture systems that maintain structural and functional characteristics in vivo using mature lung tissue cells and can be used as screening systems for drug-induced lung injury in the drug development stage. In addition, an air–liquid interface culture (ALI) was used for airway stem cell differentiation into multiciliated cells (Fulcher and Randell 2012). Currently, brain-dead donor-derived lungs are the major sources of lung primary cells, which cannot be used for the transplantation of surgically resected lung tissue of patients with early stage lung cancer. Owing to the nature of these sources, it is often difficult to obtain large amounts of healthy donor lung tissues. Recently, researchers have reported on a method to obtain and expand cells and generate airway organoids, even from lung lavage fluid samples (Sachs et al. 2019). Minimally invasive primary cell collection and organoid culture may facilitate determining the effect of drugs or side effects on individual patients (Huch et al. 2015; Broutier et al. 2017; Li et al. 2018; Berkers et al. 2019; Boretto et al. 2019). In addition, organoids derived from non-small cell lung cancer tissues retain tumor characteristics and respond differently to molecular-targeted anti-tumor drugs, depending on the genetic background of the patient (Sachs et al. 2019; Kim et al. 2019). Therefore, there is an increasing potential to use the benefits of primary cells to evaluate compounds that consider the genetic background of individual patients. Furthermore, primary lung tissue cells can be expanded and cryopreserved (Katsura et al. 2020). The combination of these technologies may be helpful in the creation of pulmonary disease cell panels and the comparative analysis of organoid phenotypes derived from patient-derived primary cells to understand pathological conditions and construct drug screening systems (Burridge et al. 2016; Knowles et al. 2018). The establishment of a drug evaluation system using primary cells is critical from the viewpoint of cell maturity and individual treatment. Further robust methods of generating primary-derived organoids are expected to advance the research on the etiology and therapeutic mechanisms not only for genetic diseases but also for sporadic diseases (Kondo et al. 2013; Mertens et al. 2015). However, the use of primary cells for efficacy and toxicity assessment in the early stages of drug development is challenging. Despite the need of a homogeneous cell population for high-throughput screening, primary cell-derived organoids are often a mixture of multiple cell types. In addition, the cell ratio of organoids may differ depending on the organoid preparation method and sample conditions. Variances of sites of cell origin and batches of cell preparation might need to be resolved for standardization. Furthermore, it is not realistic to obtain a cell source that is sufficient for high-throughput screening. Human PSC-derived lung cells can possibly provide a solution to the problems of in vitro assessment systems for the early stages of drug development (Table 2). Human PSCs have the potentials for reducing batch-to-batch and lot-to-lot differences thereby enabling large-scale drug screening because they can be cultured in large numbers while maintaining an undifferentiated state. Moreover, their reporter cells can be used for the enrichment of the target cell population. In recent years, several methods for generating efficient and scalable lung epithelial cells from human PSCs have been reported, and the implementation of drug screening using PSC-derived lung epithelial cells has become a probable choice (Fig. 2).

**Table 2:** Comparison of the characteristics of iPS cell-derived and primary cells

	Advantages	Disadvantages
Primary cells	Mature cells Donor specific genetic background (Probably) maintained epigenetic background (Probably) maintained acquired features	Limited source Limited number of passages invasiveness Hard to serve a donors’ panel difficult cloning
iPS cells	Easy to obtain High expandability Accessible to large number of donors Potential to industrialize Easy cloning Easy genetic modification	Immature cells Multi-steps required for differentiation Expensive to maintain

### Lung bud/bud tip organoid

Lung bud/bud tip organoids recapitulate the lung bud state during embryogenesis. Human PSCs can be differentiated into the endoderm and subsequently into the anterior foregut (AFE), the origin of lung bud cells via simultaneous inhibition of transforming growth factor-β and bone morphogenic protein (BMP) signaling (Green et al. 2011). In addition, researchers developed an approach to recapitulate lung development from AFE in vitro. Two groups have reported on lung bud/bud tip organoids derived from human PSCs. These are characterized by a lung-like branching structure and a transcriptional profile similar to that of the fetal human lung, such as the presence of NKX2.1 + SOX9 + SOX2 + cells (Chen et al. 2017; Miller et al. 2018). Lung bud/bud tip organoids reportedly include both alveolar and airway epithelium (Chen et al. 2017; Miller et al. 2018). Therefore, they could be used as a system to evaluate the effects of drugs on development as well as on epithelial cells following differentiation. Snoeck and colleagues also evaluated their use for disease modeling of HPS, which gets complicated by pulmonary fibrosis. They established HSP1 gene KO human PSCs to detect branching abnormalities. They also demonstrated that human PSC-derived lung bud organoids could be used as a model for respiratory syncytial virus infection studies. These phenotypic features suggest the possibility of using this system as a drug screening platform for treating genetic lung diseases and respiratory infections. Lung bud organoids are characterized by their ability to reproduce cells of the fetal lung in several aspects. Spence et al. pointed out their potential to predict toxicity in pregnancy (Miller et al. 2019). However, the degree of maturation is insufficient at both cellular and structural levels, thereby limiting their application to general drug assessment systems (Metzger et al. 2008; Alanis et al. 2014). In addition, the structural complexity of lung bud organoids requires additional efforts to detect their phenotype. Several wells should be rapidly processed during early drug screening, thereby necessitating optimization, such as the creation of appropriate reporter cells. In addition, the inclusion of multiple cell types complicates the analysis of drug-induced lung injury. It may be effective to use cells differentiated into a specific epithelial cell lineage to increase the throughput and make the system more efficient in analyzing drug-induced lung injury.

### Human PSC-derived alveolar epithelial cells

Injury of alveolar epithelial cells has been considered to cause interstitial pneumonia (Katzen and Beers 2020). Type 2 alveolar epithelial cells are a major cell type in alveoli and have stemness and secretory function and contribute to gas exchange via differentiation into type 1 alveolar epithelial cells. Therefore, it is important to evaluate toxicities of drugs on these cellular components. An expandable source of type 2 alveolar epithelial cells is necessary for the evaluation of drug efficacy and lung toxicity. However, primary type 2 alveolar epithelial cells have low proliferative potential in vitro and easily differentiate into type 1 alveolar epithelial-like cells on being isolated from human lungs and cultured in plastic plates (Borok et al. 1998; Foster et al. 2007). The aforementioned culture difficulties are a barrier to in vitro assays using alveolar type 2 epithelial cells. Several groups have reported on methods for the long-term culture of human PSC-derived type 2 alveolar epithelial cells (Yamamoto et al. 2017; Jacob et al. 2017). Type 2 alveolar epithelial cells are induced from NKX2.1-positive lung progenitor cells (LPCs), similar to other lung epithelial cells (Zorn and Wells 2009; Morrisey and Hogan 2010). The step-wise differentiation of human PSCs into the definitive endoderm and NKX2.1 + lung bud cells by a combination of BMPs, fibroblast growth factors (FGFs), wingless/integrated, and retinoic acid is widely accepted (Chen et al. 2010; Green et al. 2011). Each group has a different strategy for producing alveolar epithelial organoids from LPCs. Kotton et al. differentiated NKX2.1-positive cells (or CD47hi/CD26lo cells) into SFTPC + type 2 alveolar epithelial cells by embedding them in Matrigel and culturing them in medium containing CHIR, keratinocyte growth factor (KGF), dexamethasone, cyclic adenosine monophosphate, and 3-isobutyl-1-methylxanthine (CK + DCI) (Gonzales et al. 2002). This organoid culture system does not require mesenchymal cells and maintains its self-proliferation and differentiation potential as AT2 cells, despite prolonged passaging and cryopreservation (Hawkins et al. 2017; Jacob et al. 2017). Researchers also sorted NKX2.1-positive lung lineage cells with carboxypeptidase M (CPM), a purifiable cell surface marker, and cultured them in a medium containing CHIR99021, FGF10, KGF, and *N*-[*N*-(3,5-difluorophenacetyl)-l-alanyl]-*S*-phenylglycine *t*-butyl ester (DAPT) to polarize their lineage to the terminal epithelium (distalization step). Subsequently, they co-cultured CPM hi cells and human fetal pulmonary fibroblasts in Matrigel to differentiate into SFTPC + type 2 alveolar epithelial cells (Yamamoto et al. 2017). This method requires human fetal pulmonary fibroblasts; however, a high SFTPC + cell rate (approximately 50%) was achieved in the early period of organoid culture. The presence of AGER + PDPN + HOPX + HT1-56 + human induced PSC (iPSC)-derived AT1 cells (iAT1 cells) was also confirmed under 3D culture conditions (Kanagaki et al. 2021a). Subsequently, LPCs can be differentiated into AT2 cells by culturing under ALI conditions (Huang et al. 2020; Bluhmki et al. 2021). Applications using alveolar epithelial cell organoids have been reported for their use in disease modeling and drug toxicity assessment. Jacob et al. demonstrated that type 2 alveolar epithelial cells derived from SFTPB mutant patient-specific iPSCs revealed lamellar body failure because of the abnormal production of surfactant proteins (Jacob et al. 2017). Furthermore, they constructed a monolayer epithelial culture system using iPSC-derived alveolar epithelial type 2 cells (iAEC2) and demonstrated its application to SARS-Cov-2 infection studies. Transcriptome analysis revealed the driving inflammatory program in infected iAEC2 and the phenotype of the NF-κB-mediated inflammatory response (Huang et al. 2020), thus indicating iAEC2 is useful for evaluating drug efficacy and cytotoxicity. In contrast, the aforementioned method as well as fibroblast-dependent alveolar organoid culture (Yamamoto et al. 2017) have the limitation of low to medium throughput, and are unsuitable for analyzing numerous compounds. iAEC2 has the advantage of scalability, which enables the preparation of several pure iAEC2 cells. The construction of high-throughput readout systems for analyzing cell damage and/or functions will expand applications. Yamamoto et al. reported on giant lamellar bodies and abnormal lipid accumulation in iAT2 cells treated with amiodarone (Yamamoto et al. 2017; Kanagaki et al. 2020), an antiarrhythmic drug that causes drug-induced lung injury (Bedrossian 1997). It was quantitatively evaluated using LysoTracker, a fluorescent probe incorporated into lamellar bodies. Moreover, Korogi et al. and Suezawa et al. reported on a similar phenotype in iAT2 cells derived from each patient with Hermansky-Pudlak syndrome type 1 and 2 (Korogi et al. 2019; Suezawa et al. 2021a). The 3D co-culture of mesenchymal and epithelial cells in Matrigel may facilitate the recapitulation of epithelial-mesenchymal interactions in pulmonary fibrosis. Suezawa et al. delineated a pulmonary fibrosis model with alveolar organoid contraction caused by activated epithelial-mesenchymal interactions upon BLM treatment (Suezawa et al. 2021b). This phenotype can be evaluated by image analysis and may have a higher throughput readout than conventional evaluation systems. Hekman et al. recently reported on the screening of antiviral drugs against SARS-Cov-2 using iAEC2 expanded in organoids derived from human iPSCs (Hekman et al. 2020). They infected ALI-cultured iAEC2 with SARS-Cov-2 virus and evaluated the infection rate by immunohistochemistry for SARS-Cov-2 virus protein. They used the aforementioned method to screen 31 small-molecule compounds to inhibit viral infection. The number of compounds assessed was limited by the throughput of the assessment and/or the culture method, despite multiple compounds displaying different antiviral activity among those using Vero E6 cells and iAEC2. These results support the concept that hPSC-derived lung cells may be a promising tool for screening compounds for respiratory diseases and toxicity.

### PSC-derived airway epithelial cells

Airway epithelial cells are exposed to a variety of substances, including airborne toxins. Injury to airway epithelial cells often exacerbates diseases such as COPD and asthma (Decramer et al. 2012; Guarnieri and Balmes 2014). It is useful to evaluate toxicity of chemical agents and environmental substances for functional airway epithelial cells. In addition to alveolar epithelial cells, there are reports on several methods for differentiating airway epithelial cells. Wong et al. demonstrated that FGF7, FGF10, BMP4, and FGF18 are required to differentiate hPSC-derived endodermal cells into airway epithelial lineage cells, and that ALI culture can be adapted to induce airway epithelium (Wong et al. 2012). These cells expressed the functional halogen ion channel cystic fibrosis transmembrane conductance regulator (CFTR). Furthermore, this function was abolished in cells derived from PSCs generated from patients with cystic fibrosis harboring CFTR mutations. Subsequently, Firth et al. reported that the addition of DAPT, a Notch signaling inhibitor, contributes to the maturation of airway epithelial cells and successfully induces multiciliated cells (Firth et al. 2014). Thereafter, Konishi et al. reported on a method for inducing proximal airway epithelial progenitor cells (PAEPCs) using 3D organoids (Konishi et al. 2016). PAEPCs were efficiently differentiated into multiciliated airway cells (MCACs) in 3D organoids. In ALI cultures of human PSC-derived airway cells, multiciliated airway cells were demonstrated to functionally move similar to mature airway epithelial cells. In particular, the ciliary beat frequency and mucociliary transport were comparable to those of primary airway cells. McCauley et al. also reported on PSC-derived airway organoids as well as a forskolin assay of a cystic fibrosis model (McCauley et al. 2017). Yin et al. reported on use of the PSC-derived airway epithelial cells for a SARS-COV-2 infection model (Yin et al. 2021). The airway epithelial cells highly expressed angiotensin-converting enzyme 2 (ACE2) and transmembrane protease, serine 2 (TMPRSS2). SARS-Cov-2 infected them and activates interferon responses. Another method has been recently reported for generating airway epithelial cells. Basal cells are stem cells present in the airways, and differentiate into multiciliated cells and secretory cells following an injury to the airways. Hawkins et al. reported on a method for efficiently inducing basal cells from human PSCs (iBCs). They successfully induced iBC cells with high expandability and the potential to differentiate into secretory cells and multiciliated cells (Hawkins et al. 2021). Furthermore, these cells were applied to the disease modeling of asthma, cystic fibrosis, and primary ciliary dyskinesia. Moreover, iBCs are likely to be applied to drug evaluation owing to the advantage of their expandability. However, above-mentioned multiciliated cells derived from the PSCs including our original method (Konishi, et al. 2016) did not display unidirectional coordination of ciliary movement, as observed in vivo. Sone et al. recently reported that microfluidic chip technology enabled the recapitulation of unidirectional coordinated ciliary beating of efficiently induced multiciliated airway cells (iMCACs) in submerged conditions without ALI culture, by applying unidirectional flow to PAEPCs during differentiation (Sone et al. 2021). The aforementioned organ-on-a-chip technology would be beneficial for performing functional assessments in vitro, similar to those of living organisms than traditional ALI culture. Mimicking the biological environment by combining suitable culture devices and mechanical stimulation may have the potential to provide a solution for the immaturity of human PSC-derived epithelial 3D organoid culture. Indeed, various airway epithelial function can be examined using PSC-derived airway epithelial cells. Extracts from PM2.5, air micro particulates, and an exposure to cigarette smoke reportedly induce a decrease in the number of multiciliated cells. Moreover, researchers have reported on structural abnormalities in the cilia (Schamberger et al. 2015; Montgomery et al. 2020). The use of human PSC-derived airway epithelium will enable an understanding of the effects of compounds and harmful airborne substances on the human respiratory tract.

### Organ-on-a-chip

In 2010, Huh et al. reported on the development of a lung-on-a-chip using a microdevice to reproduce the alveolar–capillary interface environment of the lung in vivo (Huh et al. 2010). Subsequently, numerous studies have been conducted to reproduce the spatial arrangement and microenvironment in vivo to enhance cell maturation and evaluate the functional assessment of compounds in various organ cells (Jang et al. 2013; Schamberger et al. 2015; Bhise et al. 2016; Shah et al. 2016; Zhang et al. 2016). Organ-on-a-chip technology may overcome the issue of PSC-derived cells, namely the immature state of the cells. The airway-on-a-chip system, designed to simulate biological liquid flow in vitro, increased the efficiency of cell differentiation and also improved the molecular and functional maturation of cells (Benam et al. 2016a, b; Sone et al. 2021). The use of human PSC-derived type 2 alveolar epithelial cells that can differentiate into type 1 alveolar epithelial cells may validate the gas exchange function. Recent developments in lung cell differentiation protocols and scalable culture methods have removed these limitations. Recently, there have been reports on primary alveolar epithelial cells. Jain et al. demonstrated that epithelial-endothelial interactions are required in LPS-induced pulmonary thrombosis by refluxing human whole blood into lung-on-a-chip with human primary epithelial cells and HUVECs (Jain et al. 2018). Co-culture systems of endothelial cells and epithelial cells have been widely used in lung-on-a-chip systems. Principally, they used cell lines because of the difficulty in maintaining primary epithelial cells. Researchers have also conducted disease modeling and therapeutic drug identification (Huh et al. 2012). By reproducing and evaluating the cell function in vitro, the lung-on-a-chip technology can improve the accuracy of predicting drug-induced lung injury in vivo. However, it remains controversial if cell line studies can accurately mimic in vivo responses. The application of PSC-derived pulmonary epithelial cells in these systems may eliminate the limitations associated with the use of cell lines in challenging primary cell-adapted systems. Currently, human PSC-derived lung epithelial cells have improved their expandability and the maintenance of function. They may be applied to conventional and/or new lung-on-a-chips. In addition, matching the genetic backgrounds of all cell types that compose the organ-on-a-chip may enable the development of more accurate pathological models and personalized models to evaluate the toxicity of various compounds and therapeutic effects of drugs (Kudoh et al. 2008; Shi et al. 2014). Despite this possibility being previously noted (Ronaldson-Bouchard and Vunjak-Novakovic 2018), establishing robust differentiation protocols for specific cell lineages has been a critical issue, particularly in the field of human PSCs’ applications.

### Co-culture system

A characteristic feature of organoid culture systems is the co-culture state of multiple cell types. In a biological environment, multiple types of cells interact with each other to perform organ functions as a unit. In the PSC-derived organoid culture system, researchers are attempting to create more functional cells by mimicking cell–cell interactions in vivo. Yamamoto et al. reported on the co-culture of alveolar epithelial organoids and fibroblasts to differentiate into type 2 alveolar epithelial cells and the subsequent study demonstrate that type 1 alveolar epithelial cells were derived from type 2 alveolar epithelial cells (Yamamoto et al. 2017; Kanagaki et al. 2021a). Furthermore, they described fibrotic phenotypes caused by epithelial-mesenchymal interactions. Mesenchymal and immune system cells regulate lung tissue regeneration by the proliferation and differentiation of type 2 alveolar epithelial cells (Westphalen et al. 2014; Lindemans et al. 2015; Lechner et al. 2017). Choi et al. reported that the optimal regulation of IL-1b signaling affects the formation and proliferation of type 2 alveolar epithelial organoids from mouse primary cells co-cultured with interstitial macrophages. Furthermore, IL-1b signaling is important for the generation of differentiation intermediates that occur following BLM injury and their normal differentiation into type 1 alveolar epithelial cells (Choi et al. 2020). However, these studies were performed on mouse cells, and it is unclear if similar phenomenon can be observed in humans. Studies using human PSC-derived alveolar epithelial cells and lymphocytes may clarify this issue. Drug-induced lung diseases are accompanied by cytotoxic and allergic reactions involving immune system cells (Cooper et al. 1986a, b). In addition, although the relationship between lung epithelial cells and immune cells in immune checkpoint inhibitors remain to be elucidated, it might play a central role in various diseases, including interstitial pneumonia. The co-culture system using human PSC-derived lung epithelial cells has the potential to detect recent drug-induced lung injury. Drug lymphocyte stimulation test is currently used in clinical practice and has several limitations, such as specificity (Pichler and Tilch 2004). Co-culture with human PSC-derived airway or alveolar epithelial cells may contribute to solving the aforementioned problems for predicting drug-induced lung diseases.

### Materials for cell culture

The cell culture matrix is an important issue in the study of PSC-derived lung epithelial cells. The major factors to be considered include components of the extracellular matrix (ECM) and 3D organization. Currently, most 3D lung cell organoids are embedded in Matrigel, a solubilized basement membrane preparation extracted from the Engelbreth–Holm–Swarm mouse sarcoma. It supports the differentiation, maintenance, and functioning of cells in numerous organs by providing scaffolds with a biomimetic ECM composition and appropriate plasticity (Lancaster and Knoblich 2014). In contrast, the Matrigel comprises several undefined mouse-derived proteins, in addition to lot-to-lot and batch-to-batch differences (Hughes et al. 2010). Culturing in such an environment is a hurdle for an application in regenerative therapy, and may also influence the results of drug evaluation, including toxicity assessment. The development of scaffolds as an alternative Matrigel is underway to overcome these issues (Zimoch et al. 2018). de Carvalho et al. mentioned that the differentiation of LPCs into lung epithelial cells occurs in 3D cultures using a collagen I matrix (de Carvalho et al. 2019). Culture in the Col I matrix resulted in increased expression of greater epithelial marker molecules (AT1, AT2, airway epithelium) than that in Matrigel. Col gels may be more tolerant to cell maturation. This necessitates determining if LPCs and their derivative cells contribute to maturation when cultured in a matrix other than Matrigel. Ikeo et al. reported on the possibility to expand LPCs while maintaining their potential to differentiate into both airway and alveolar lineage cells by seeding LPCs in sodium alginate nanofibers. Type 2 alveolar epithelial cells differentiated in microfibers demonstrated higher expression levels of type 2 alveolar epithelial cell marker genes than conventional cultures that produce alveolar organoids in Matrigel. Revisiting traditional 3D arrangement may improve cell maturation Matrigel may not be necessary for the differentiation and maintenance of PSC-derived lung epithelial cells. The use of appropriate materials may also provide a solution to the immaturity of cells, a problem in several culture systems. The improvement of cell maturation, the reduction of lot-to-lot and batch-to-batch differences, and the improvement of handling supposedly realized by switching away from Matrigel will also enable the assessment of compounds. Moreover, 3D cell layouts and contacts have a significant impact on the behavior and state of cells. 3D bioprinting technology is widely used to create cells that are more similar to biological bodies and to construct culture systems that are closely related to biological functions (Stevens and George 2005; Murphy and Atala 2014; Moldovan et al. 2017; Duval et al. 2017). Repositioning the current human PSC-derived airway and alveolar organoids with a 3D bioprinter may lead to more mature and/or functional improvements. Researchers have also performed recellularization studies, in which the cells are seeded onto scaffolds devoid of cellular components from living lungs (Taylor et al. 2018). In PSC-derived lung cells, Ghaedi et al. seeded type 2 alveolar-like cells differentiated from PSCs onto decellularized rat lung or human alveolar tissue sections. The seeded cells reportedly maintained their own characteristics and differentiated into type 1-like alveolar epithelial cells (Ghaedi et al. 2013). If the scalability of iAT2 cell production improves, we will be able to screen potential drugs that cause differentiation abnormalities in the regeneration of the reconstructed lung microenvironment.

### In vivo humanized model

It is difficult to predict drug efficacy and drug-induced lung injury in clinical practice using current in vivo evaluation systems owing to species differences. The transplantation of human PSCs into mice may solve this problem. Liver bud organoids and intestinal organoids can be transplanted into mice with damaged organs (Yui et al. 2012; Takebe et al. 2013). The transplanted human cells were incorporated into mice organs and displayed organ-like functions. Furthermore, researchers have detected human-specific metabolites in the mouse liver (Takebe et al. 2013). In other words, it may be possible to estimate the drug response in humans in vivo using mice with humanized organs derived from human PSC cells. Miller et al. reported on the successful engraftment of human PSC-derived lung tip bud organoid cells in the airways of naphthalene-treated mice and the differentiation of airway epithelial cells (Miller et al. 2018). Ikeo et al. transplanted human PSC-derived LPCs into naphthalene-and radiation-treated lung-injured mice. The transplanted LPCs were engrafted in mouse lung tissue and differentiated into type 2 alveolar epithelial cells and airway epithelial cells in vivo, as demonstrated by transcriptome analysis (Ikeo et al. 2021). The use of humanized mice with human lung cells may result in a more biologically relevant evaluation of drug efficacy and pulmonary toxicity.

## Discussion

It is difficult to accurately predict in vivo assessments of drug efficacy and lung injury in humans because of species differences. In addition, it is difficult to analyze the exact mechanism of drug-induced lung injury that occurs in the clinical environment caused by multiple factors, such as kinetics in vivo. Human PSC-derived lung-related cell culture systems are currently under research and development, and may overcome these issues. In vitro culture systems that mimic living organisms, such as organoids and/or organ-on-a-chip cultures, various factors and conditions can be set for evaluation. Moreover, researchers can select combinations of multiple lineage cells, such as epithelial and mesenchymal cells. These features are beneficial for understanding the mechanisms of pulmonary drug toxicity in future. The rapid identification of the mechanism of new drug-induced lung injury in clinical practice will enable the development of a method to avoid or prevent it, besides eventually maximizing the clinical and economic benefits and minimizing the risks of drugs. Furthermore, considering the ideal human cell culture system, we would be able to demonstrate the efficacy and safety of candidate therapeutic agents that have previously failed in animal studies. We may be able to avoid the exclusion of promising candidate therapeutics. The present culture systems have been able to capture various functional phenotypes. These results will be useful for the analysis of the mechanism of drug-induced lung injury. In addition, recent advances in genome editing technologies, such as CRISPR and omics analysis at single-cell level resolution, such as single-cell RNA-seq and ATAC-seq, will make PSC-based methodology more attractive for analyzing or predicting human biological responses. However, researchers have reported on few cases of large-scale screening with lung cell organoids (Han et al. 2020). This can be primarily attributed to poor throughput. Recent advances in the long-term expansion of hPSC-derived lung cells have been remarkable for alveolar stem cells (Yamamoto et al. 2017; Jacob et al. 2017), distal LPCs (Chen et al. 2017; Miller et al. 2018; Ikeo et al. 2021), and basal stem cells (Hawkins et al. 2021). It is now possible to secure numerous LPCs and their derivative lung epithelial cells in a robust and reproducible manner. A bottleneck is required for organoid formation and phenotype detection. It is also essential for HTS-using lung cell organoids to realize a high-throughput method to not only assess cell death but also features and functional outputs that are specific to the lung epithelium. Image analysis is considered an effective and high-throughput readout. Self-organization is one of the characteristics of organoids, and changes in organoid morphology have been reported in several disease modeling studies (Chen et al. 2017; Alysandratos et al. 2021a). Human PSCs can be easily gene-edited, and it is possible to generate injury marker reporter cell lines. Machine learning and deep learning are well suited for analyzing such visual output. In addition to histological images, they can capture few features of the tissue surface and discriminate the diseased tissue from normal tissue at an incredible speed (Sakai et al. 2018; Rajpurkar et al. 2018; Akkus et al. 2019). Furthermore, there have been reports on the analysis of changes in the size and shape of three-dimensionally arranged organoids upon differentiation by machine learning (Kassis et al. 2019; Kegeles et al. 2020). A combination of these technologies to perform a high-throughput evaluation of compound toxicity and drug efficacy based on visual changes would be a strong tool to enable compound screening in the early stage of drug development. In addition, the toxicity of drugs that have caused problems in clinical situations or have been dropped during development should be evaluated using the latest culture systems, including organoids. The accumulation of knowledge on the in vitro phenotypes of compounds with known clinical problems in functional lung cell culture systems is essential for improving the accuracy of compound toxicity estimation. Such advances will facilitate a comprehensive analysis of known lung injury-inducing agents. Similar to other organ organoids, lung organoids, including human PSC-derived organoids, have overcome several obstacles to their implementation. Currently, culturing methods with excellent expandability have been established, and the maturity of cells has been improved in both molecular and functional aspects. In future, pathological analysis and drug screening methods using human PSC-derived lung organoids will be continually developed depending on the miscellaneous purposes, including toxicology in vitro and in vivo.
